# Nano- and Microplastics in the Cardiovascular System: Current Insights and Biological Implications

**DOI:** 10.3390/nano16100589

**Published:** 2026-05-12

**Authors:** Mario Cristina, Manuel Belli, Anna Baroni, Chantalle Moulton, Emily Carinci, Marta Gatti, Ennio Tasciotti, Matteo Antonio Russo, Patrizia Russo, Luigi Sansone

**Affiliations:** 1 Laboratory of Molecular, Cellular and Ultrastructural Pathology, IRCCS San Raffaele Roma, 00166 Rome, Italy; manuel.belli@uniroma5.it (M.B.); matteo.russo@sanraffaele.it (M.A.R.); luigi.sansone@uniroma5.it (L.S.); 2 Department of Human Sciences and Promotion of the Quality of Life, San Raffaele Roma University, 00166 Rome, Italy; emily.carinci4@gmail.com (E.C.); ennio.tasciotti@sanraffaele.it (E.T.); patrizia.russo@uniroma5.it (P.R.); 3 Human Longevity Program, IRCCS San Raffaele Roma, 00166 Rome, Italy; anna.baroni@sanraffaele.it (A.B.); chantalle.moulton@sanraffaele.it (C.M.); 4 Department of Medicine, Rheumatology and Clinical Immunology, University of Rome Campus Bio-Medico, 00128 Rome, Italy; marta.gatti92@gmail.com; 5 San Raffaele Sulmona, Viale dell’Agricoltura, 67039 Sulmona, Italy; 6 Clinical and Molecular Epidemiology, IRCCS San Raffaele Roma, Via di Val Cannuta 247, 00166 Rome, Italy

**Keywords:** microplastics, nanoplastics, cardiovascular diseases, cardiotoxicity, endothelial dysfunction, cardiomyopathy, inflammation, rehabilitation

## Abstract

Micro- and nanoplastics (MNPs) are ubiquitous environmental pollutants recognized as emerging and relevant risk factors for numerous human diseases, including cardiovascular diseases. MNPs enter the human body through ingestion, inhalation, and dermal penetration, and their toxicity varies according to size, shape, and chemical composition, most notably between microplastics (>1 µm) and nanoplastics (<1 µm), which differ in cellular uptake mechanisms and biodistribution. Recent evidence has confirmed their presence in cardiac and vascular tissues, raising significant concerns about their potential impact on human health. This review summarizes current knowledge on MNP exposure sources, physicochemical properties, and systemic bioavailability, with a particular emphasis on the mechanisms of transport that facilitate their deposition within the myocardium and vasculature. It further addresses a broad spectrum of cardiotoxic effects, including oxidative stress, mitochondrial injury, immune activation, ion channel disruption, cell death, and fibrosis. Endothelial dysfunction, vascular injury, and pro-atherogenic activity are also discussed. In addition to outlining existing detection techniques and emerging in vitro models, the review highlights initial steps toward the development of preventive strategies. Concluding with key knowledge gaps and future research directions, this article underscores the urgent need for standardized measurement tools, deeper insights into damage mechanisms, and clinical interventions to prevent MNP-induced cardiovascular diseases.

## 1. Introduction

**Figure 1:**
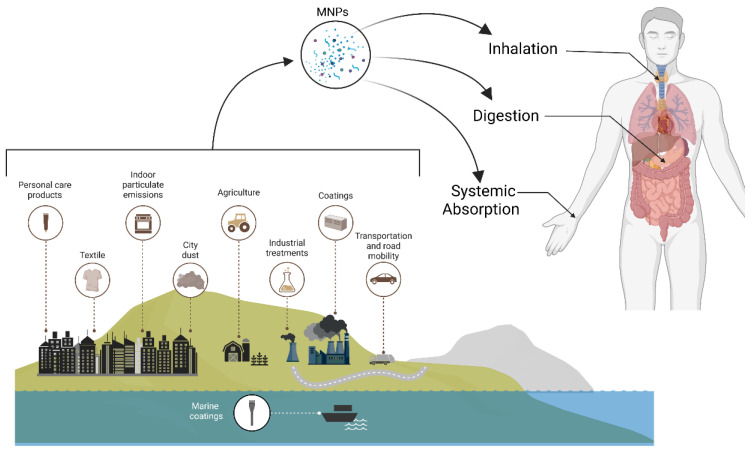
Environmental sources and human exposure routes of MNPs. MNPs arise from environmental sources such as degraded plastic waste, industrial production, and consumer products. They are present in air, water, soil, and food, leading to human exposure primarily through ingestion, inhalation, and systemic absorption, for example, via dermal contact.

Plastics are man-made polymers and, currently, an essential part of contemporary living because of their resistance, flexibility, and simplicity in production. However, they have become unregulated pollutants in nature as microplastics (MPs, 1 µm up to 5 mm) and nanoplastics (NPs, <1 µm) [1]. They are either produced by the fragmentation of larger plastic pieces or chemically prepared for commercial and consumer applications. Micro- and nanoplastics (MNPs) have now been identified in drinking water, diet, air, and human tissues, with chronic exposure through ingestion, inhalation, and dermal permeation raising concerns (Figure 1) [2,3].

In recent years, the focus has shifted from the highly publicized gastrointestinal and respiratory effects of MNPs towards their putative role in cardiovascular disease. Cumulative experimental and clinical evidence demonstrates that MNPs can traverse biological barriers, enter the circulatory system, and are deposited in vascular and cardiac tissues, where they can induce endothelial dysfunction, oxidative stress, mitochondrial damage, inflammation, and fibrosis [4,5]. Despite ongoing evidence, the overall cardiovascular toxicity and long-term effects of MNP exposure are yet to be established, and some mechanisms remain controversial [6,7]. Furthermore, plastic micro-additives like bisphenols and phthalates contribute to cardiovascular dysfunction by disrupting endocrine function and altering adipose tissue regulation [8,9]. Considering the worldwide extent of plastic pollution and the increasingly available data connecting MNP exposure to adverse cardiovascular effects, a thorough understanding of their function is an immediate need. The purpose of this review is to summarize knowledge regarding environmental exposure to MNPs, their physicochemical properties, and mechanisms of transport and accumulation in the cardiovascular system. It further explores toxicological mechanisms, highlights vascular and myocardial health effects, and discusses novel therapeutic targets and prevention strategies. Although several recent reviews have examined the cardiovascular effects of MNPs [3,10,11,12], most have primarily provided descriptive overviews of toxicity, focused on specific experimental models, or addressed individual mechanisms in isolation. For instance, previous works [3,10] provide broad overviews of exposure routes and systemic toxicity, while others [11] present a structured scoping review summarizing current clinical and experimental evidence. Previous mechanistically oriented reviews focus specifically on mitochondrial dysfunction [12], whereas others [13] emphasize the possible clinical aspects. Despite these advances, these studies generally address isolated aspects of MNP toxicity.

In contrast, the present review aims to offer a comprehensive and integrative perspective that bridges environmental exposure, systemic distribution, and cardiovascular-specific pathophysiology within a unified framework. Particular emphasis is placed on the role of mitochondrial dysfunction as a central mechanistic hub linking oxidative stress, inflammatory activation, and multiple regulated cell death pathways. By integrating mechanistic insights with translational approaches, including advanced in vitro models such as organoids and organ-on-chip systems, this review aims to provide a more consistent understanding of how MNPs may contribute to cardiovascular disease and to identify key gaps for future research.

## 2. Micro- and Nanoplastics as Emerging Pollutants: Biological Evidence and Analytical Approaches

MNPs, which include microplastics and nanoplastics ranging in size from 1 µm to 5 mm, are currently ubiquitous environmental contaminants due to the boom in plastic production and the uncontrolled disposal of plastic waste. MNPs now dominate almost all environmental ecosystems, aquatic, freshwater, terrestrial, and aerial, and have significant ecological and human health impacts [14,15,16]. Their high dispersion, stability, and chemical stability make them highly resistant to the metabolism of biological organisms and environmental processes. MNPs are heterogeneous in their origin, chemical composition, and structure. They are either primary MNPs, which are produced intentionally (e.g., dishwasher detergent with microbeads), or secondary MNPs, which are degraded from plastic items such as packaging, clothes, or fishing nets. Material-wise, the most prevalent polymers are polyethylene (PE), polypropylene (PP), polystyrene (PS), polyethylene terephthalate (PET), and polyvinyl chloride (PVC). They exist in numerous shapes, such as spheres, fibers, pellets, fragments, and films, which impact their environmental uptake and fate. MNPs consist of various toxic chemicals such as heavy metals, polycyclic aromatic hydrocarbons (PAHs), persistent organic pollutants (POPs), and antibiotics. If ingested, these toxins can enter the body and cause widespread systemic toxicity [17,18,19]. MNPs are readily absorbed by aquatic and freshwater organisms, resulting in their bioaccumulation and biomagnification throughout the food chain [3,14]. Their presence with co-contaminants can cause synergistic or inhibitory toxicities, increasing the difficulty of quantifying human health risk [20,21]. In addition to environmental persistence, epidemiologic data also quantify human exposure. A cross-sectional study recently identified MNPs in 77% of human blood samples analyzed, and some studies identified MPs in lung tissue, the placenta, feces, and breast milk. Although the source remains unknown to date, the evidence is reminiscent of widespread and long-term exposure. Correlations between the presence of MNPs and systemic inflammatory markers, oxidative stress, vascular disease, and metabolic disturbances associated with chronic health disturbances, such as cardiovascular and endocrine disease, have been established [3,8]. Geographically, human beings in very industrialized or urban settings, especially in low and middle-income nations with poor waste management, are subjected to heightened exposure levels. Vulnerable populations, for instance, infants, elderly people, pregnant women, and individuals with pre-existing cardiometabolic illness, may suffer increased risk due to physiological vulnerability and the high load of pollutants. In addition to their possible toxicity, MPs also disrupt fundamental ecological processes. They destabilize microbial food webs in terrestrial and aquatic ecosystems, suppress nutrient cycling, and adversely affect crop yields and animal health in agroecosystems [22,23]. Despite this intrinsic environmental and compositional diversity, a substantial proportion of experimental studies investigating MNP toxicity rely predominantly on monodisperse polystyrene (PS) particles as model systems. While these particles offer clear advantages in terms of reproducibility and experimental standardization, their environmental relevance remains debated. In natural environments, MNPs are characterized by heterogeneous mixtures of polymers, irregular shapes, and weathered surfaces often associated with biofilms and adsorbed contaminants [24]. In contrast, commercially available PS beads are typically uniform, spherical, and chemically simplified, which may limit the extrapolation of experimental findings to real-world exposure scenarios and contribute to an oversimplified interpretation of toxicity mechanisms. The final environmental fate, transport routes, and synergistic toxicity of MNPs with co-pollutants remain poorly understood, underscoring the need for coordinated detection protocols, hybrid research designs, and effective environmental and public health interventions [14,21].

### 2.1. Differences Between Microplastics (>1 µm) and Nanoplastics (<1 µm) in Cellular Uptake and Toxicity

MNPs may vary significantly in their interaction with biological systems due to differences in size, surface area, and physicochemical attributes, as well as in their biological behavior, such as interactions with barriers and cells, uptake, intrinsic activity, and toxicity. NPs, by virtue of their nanoscale size, are internalized far more readily through mechanisms such as clathrin-mediated endocytosis, macropinocytosis, and even passive diffusion [25,26]. While MPs are generally retained in extracellular spaces or internalized via endocytic or phagocytic pathways and subsequently compartmentalized within endosomal or phagosomal vesicles, NPs reach deeper into the cell, accumulating within organelles like mitochondria, lysosomes, and nuclei, thereby disrupting critical cellular functions [27]. This heightened internalization explains their heightened cytotoxicity. NPs have also been found to induce oxidative stress, mitochondrial damage, inflammation, apoptosis, and even genotoxicity, in some instances, even after exposure to lower doses of MNPs [27,28]. They possess a larger surface-area-to-volume ratio, because of which they interact more vigorously with cellular membranes, receptors, and proteins capable of provoking immune responses and disrupting intracellular signaling pathways [29]. In addition, NPs are effective delivery systems for harmful environmental toxins such as heavy metals, PAHs, and endocrine disruptors. They can increase the toxicity of such chemicals once inside the body [28,30,31]. They can also pass more readily across biological barriers such as the intestinal epithelium and the blood–brain barrier compared to larger particles [32]. Collectively, while NPs and MPs are a threat to human health, evidence suggests that NPs produce stronger systemic effects through their enhanced permeability, intracellular targeting, and molecular interference. These differences justify the treatment of these particles as a unique and possibly more dangerous class of plastic pollution.

### 2.2. Clinical Evidence of the Presence of Micro- and Nanoplastics in Cardiac and Vascular Tissue: Clinical Findings

Until recently, much of what we know about MNP exposure and cardiovascular risk has been derived from experimental models or from circumstantial observations. Recent clinical research is now providing direct evidence that these particles are not simply environmental contaminants but are present within human cardiovascular tissues. The ground-breaking research in the New England Journal of Medicine established that polyethylene and polyvinyl chloride particles were present in about 60% of carotid artery plaques among patients undergoing endarterectomy. Interestingly, individuals with these plastics in their plaques were nearly 4.5 times more likely to experience a myocardial infarction, stroke, or death during a three-year follow-up compared to those without such particles [33]. These results, while not demonstrating causation, show a disturbing correlation between MNPs and adverse cardiovascular outcomes. This, as documented in a recent scoping review, MNPs have been identified in an assortment of human cardiovascular samples, such as venous blood, thrombi, and atherosclerotic plaque, again attesting to the fact that these particles permeate the circulatory system [11,34]. In addition, multiple studies have reported cardiovascular tissue contamination, such as in plaques, saphenous veins, and whole-blood samples [13,35]. There are also initial hints from conference reports of severe MNP accumulations in symptomatic compared to asymptomatic subjects. A recent study reported that arterial plaques from stroke patients contained significantly higher levels of MNPs than those from healthy control subjects. Moreover, plastic-filled plaques exhibited variable gene expression patterns in cells, particularly related to inflammation and plaque stability [33]. These clinical findings provide a disturbing picture: MNPs are found in human cardiovascular tissue, and their presence does not appear to be coincidental. While the evidence is still emerging and largely observational, it underscores the urgent need to clarify how these particles affect cardiovascular health.

### 2.3. Detection Techniques for MNPs: Composition, Molecular, Structure, and Function Levels

The detection and characterization of MNPs require an integrated set of analytical and experimental techniques spanning multiple scales, from material properties (e.g., polymer composition and structure) to biological systems (e.g., molecular, cellular, and tissue-level interactions). Due to their nanoscale size, heterogeneous composition, polymer matrices, and complex behavior in biological environments, no single technique is sufficient, thus necessitating a comprehensive approach to compositional, structural, molecular, and functional analyses [36,37]. At the compositional level, advanced imaging and spectroscopy techniques are common. Transmission and scanning electron microscopy (TEM, SEM) [38,39], atomic force microscopy (AFM) [40,41], and mass spectrometry (pyrolysis-GC/MS and isotopic labeling) [42] allow for the visualization and identification of MNPs within tissues and complex matrices, providing essential information on particle size, morphology, elemental composition, and polymer type with high sensitivity [43]. These methods are essential in determining the existence of MNPs and distinguishing them from other particulate matter. At the molecular level, recent advances in biosensing platforms, particularly those based on noble metal nanoparticles, enable the detection of epigenetic changes, transcriptional and post-transcriptional changes, and cell proliferation and differentiation markers. Electrochemical, optical, and piezoelectric transduction platforms have been successful in the detection of minimal changes in biological systems exposed to MNPs, providing evidence for cellular-level mechanisms of toxicity [44,45,46]. Structural identification of the particles and interactions with biological systems often relies on experiments using cell-line models, high-resolution microscopy, and machine learning interfaces. Particle tracking and image segmentation algorithms are increasingly being used to facilitate automatic determination of MNP size, shape, and distribution, especially for high-throughput applications [47,48,49]. At the functional level, platforms such as organoids, 3D scaffolds, and microfluidic devices (“organs-on-a-chip”) are increasingly being used to investigate how MNPs affect tissue-level functions. These platforms can detect functional changes, such as alterations in contractility, permeability, and inflammatory responses, thereby bridging the gap between cellular observations and organ-level outcomes [50,51,52]. To determine the distribution and origin of MNPs and to distinguish between exposure sources, experimental approaches employing fluorescent, metal-based, stable isotope, or radioisotope labeling have proven highly effective. These strategies enhance the visualization of particles in in vivo and in vitro models and enable semi-automated tracking in dynamic biological systems [53]. The synergy of these approaches with machine learning, computational modeling, and high-data processing pipelines is driving the field very rapidly, enabling improved throughput, accuracy, and reproducibility in MNP studies. This multi-dimensional approach is essential for unraveling the complex behaviors and bioeffects of MNPs in cardiovascular and systemic contexts [54,55].

However, a significant limitation highlighted in the literature concerns the discrepancy between the concentrations of MNPs used in experimental studies and the levels detected in human blood to date. Biomonitoring analyses typically report concentrations on the order of ~1–2 µg/mL, whereas many in vitro studies observe biological effects only at concentrations one to two orders of magnitude higher. Although the use of elevated doses is common for identifying potential damage mechanisms, this reduces the direct relevance of the findings for human risk assessment. Moreover, comparisons based solely on mass (µg/mL) are problematic, as the number of particles and surface area can vary considerably at the same mass depending on particle size. In addition to these dose-related discrepancies, methodological concerns arise from the presence of impurities and byproducts in particle suspensions. Commercial nanoplastic preparations, particularly polystyrene particles, may contain residual additives or contaminants that can independently induce biological responses. Without appropriate controls (e.g., filtrate or cell-free assays), toxicity may be misattributed to the particles themselves, affecting the reliability and comparability of current studies [56]. This discrepancy represents one of the main obstacles to extrapolating experimental results to human health [57].

## 3. Mechanisms of MNPs Translocation, Distribution and Cardiac Accumulations

The presence of MNPs in air, water, and food is responsible for bioaccumulation in human tissues and organs. At present, it is estimated that an individual may ingest approximately 0.1–5 micrograms of MNPs per kilogram of body weight per day through food, and up to about 0.2 micrograms per kilogram via inhalation. Recent evidence indicates that human exposure to MNPs occurs at relatively low but chronic levels, with estimated daily intake ranging from approximately 553 particles for children to 883 particles for adults, leading to cumulative body burdens of 8.3 × 10^3^ particles by early adulthood and up to 5.0 × 10^4^ particles over a lifetime [56]. In contrast, many experimental studies employ substantially higher concentrations, often designed to elicit measurable toxicological responses within short timeframes. This discrepancy highlights a key limitation in directly extrapolating experimental findings to human health risk. Moreover, exposure assessment remains complicated by differences in reporting metrics. Most experimental studies rely on mass-based concentrations (e.g., mg/kg), whereas environmental and clinical studies increasingly report particle number-based metrics. Importantly, particle size, surface area, and physicochemical properties critically influence biological interactions, meaning that equivalent mass doses may not correspond to equivalent biological effects. As noted in recent reviews, smaller particles exhibit greater bioactivity and toxicity despite lower mass contributions [58]. Additionally, real-world exposure occurs as a complex mixture, with MNPs acting as carriers for co-contaminants such as heavy metals and persistent organic pollutants, which may amplify biological effects and further complicate dose–response relationships [59].

Given that, only a very small fraction of these particles is thought to effectively translocate into body tissues (less than 1%), and differences in analytical and sampling methods still make comparisons across studies difficult [60]. The bioaccumulation of MNPs occurs through different routes:

*Inhalation* is a primary entry route. These inhaled particles interact with the respiratory mucous membranes, penetrate into the lungs, and provoke inflammation and chronic irritation [61,62].

*Ingestion* is another major exposure pathway. MNPs contaminate common foods such as fruits, vegetables, and seafood. Marine pollution causes MNP accumulation in the digestive tract, gills, and tissues of edible shellfish, creating a direct contamination source [63].

*Plastic leaching.* Additionally, high levels of MNPs are found in bottled water due to plastic leaching from packaging. MNPs come into contact with the digestive tract mucosa and tissues, allowing absorption into the bloodstream [64].

*Contact.* Beyond inhalation and ingestion, MNPs can penetrate the skin through hair follicles, sweat glands, or damaged areas. Research shows that polystyrene particles up to 2 µm can pass through the epidermis and dermis in human and animal skin models. Notably, human skin is more permeable than mouse skin, probably due to larger hair follicles. Additionally, surface functional groups like -COOH or -NH_2_ improve the interaction of NPs with stratum corneum lipid membranes, further aiding penetration [65].

### 3.1. Mechanisms of MNPs Translocation and Transport in the Bloodstream

Following entry into the body, MNPs tend to accumulate at interfaces with the external environment, such as the lungs, intestines, and skin. Small particles penetrate the lungs, accumulating in the alveoli, and can be eliminated by macrophages. Furthermore, MNPs accumulate in the endolysosomes and mitochondria of epithelial cells, which subsequently release them into the bloodstream [66,67]. MNPs also enter intestinal epithelial cells via endocytosis. Their accumulation causes alterations in tight junctions, leading to local inflammatory states [68]. Moreover, MNPs in contact with hair follicles, under specific conditions such as the presence of microlesions, can penetrate the skin barrier [25]. Once in the bloodstream, nanoparticles undergo significant surface changes, creating a biomolecular corona composed of proteins and plasma lipids. The composition of this corona significantly influences the final localization of the MNPs: they can undergo opsonization by proteins, promoting their removal, or interact with lipoproteins and albumin, prolonging their circulation time [69]. In vivo studies have also shown that these particles can cross the blood–brain barrier (BBB): polystyrene nanoparticles measuring approximately 300 nm were found in the brains of mice within two hours of oral ingestion. Molecular dynamics simulations indicate that the type of corona influences this crossing. A cholesterol-rich lipid coating facilitates particle entry into the phospholipid bilayer of the blood–brain barrier, while a predominantly protein corona blocks their passage [70]. This highlights how the surface changes that occur in the bloodstream influence the ability of nanoparticles to cross selective physiological barriers and accumulate in sensitive organs.

### 3.2. Mechanisms of MNP Accumulation in the Myocardium

**Figure 2:**
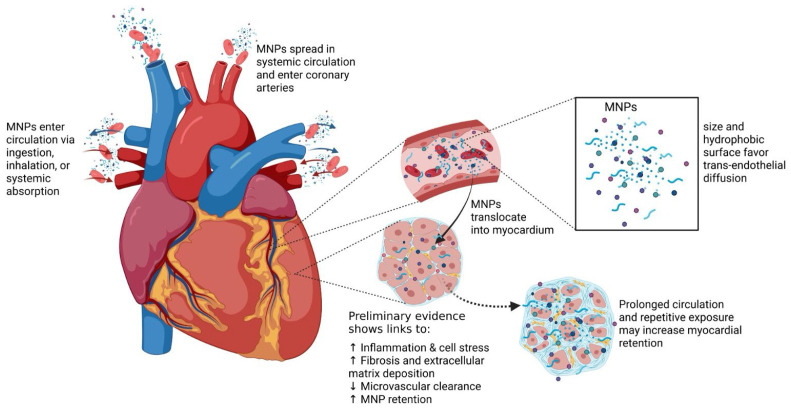
Pathway of micro- and nanoplastic (MNPs) entry and myocardial toxicity. MNPs enter the body through ingestion, inhalation, or systemic absorption, circulate bound to plasma proteins, and accumulate in the myocardium through the coronary circulation. Once inside cardiomyocytes, MNPs localize to mitochondria, causing structural damage, oxidative stress, inflammation, and apoptosis. These processes lead to fibrosis, remodeling, and impaired cardiac contractility.

MNPs, especially polystyrene (PS)-NPs, can accumulate in cardiac tissue through various exposure routes, including ingestion, inhalation, and systemic absorption. Due to their small size (<1 µm) and hydrophobic properties, they can bypass physiological barriers and spread into various cardiac tissues, including the myocardium, pericardium, pericardial fat, coronary arteries, smaller vessels, and connective scaffolds. Importantly, MNPs may directly enter cardiomyocytes, supporting their intracellular accumulation within cardiac cells (Figure 2) [71,72]. Studies of particle localization in mitochondria derived from polystyrene nanoparticles in artificial conditions showed that once inside cells, particles tend to gather in various membrane-bound organelles, such as mitochondria, suggesting a preferential subcellular distribution. Several studies indicate that mitochondrial dysfunction represents a central mechanism underlying MNP-induced cardiotoxicity. Experimental in vitro and in vivo studies consistently demonstrate the internalization of NPs into cardiomyocytes, reduction in mitochondrial membrane potential, impairment of OXPHOS complexes, decreased ATP production, and excessive reactive oxygen species (ROS) generation, ultimately promoting apoptosis, inflammation, and myocardial remodeling [73]. While these observations suggest a link between intracellular accumulation and cellular alterations, the detailed molecular mechanisms of MNP-induced cardiotoxicity are discussed in Section 4.

Mitochondrial damage can trigger molecular events like excessive ROS generation (leading to ferroptosis), activation of the inflammasome (with production of IL-6, TNF-α, and IL-1β), and the release of death signals (such as cytC, apaf, and mtDNA), causing cardiomyocyte degeneration and definitive death [12]. TEM and fluorescence imaging confirm the internalization of particles in cardiomyocytes, showing damage to mitochondrial cristae, the release of mitochondrial DNA into the cytoplasm, and the activation of a senescent response [74,75]. This damage promotes cardiac remodeling, leading to ventricular dilation, interstitial fibrosis, and decreased ejection fraction, a progression that worsens with increased doses and longer exposure [76]. Both in vivo and in vitro studies show that MNPs disrupt cellular communication by inhibiting the PI3K/AKT pathway and activating the pro-apoptotic gene BCL-2, resulting in abnormal cell death and the replacement of cardiomyocytes with fibrous tissue. Besides reducing contractile function, this process also impairs electrical conduction in the heart, raising the risk of arrhythmias, as seen in human cardiac stem cell models [77].

Other membrane-bound organelles may be affected by MNPs. It has been shown that exposure to NPs can involve lysosomes, molecular condensates or P-granules, multivesicular bodies, and phagolysosomes, suggesting interference with intracellular trafficking and degradation pathways. Further, MNPs may trigger endoplasmic reticulum stress, leading to intracellular protein denaturation or misfolding, with relevant effects on cardiac metabolism and dramatic loss of cytosolic Ca^2+^ homeostasis [10]. In addition, changes in mitochondrial potential and the inhibition of biosynthetic pathways (Krebs cycle and OXPHOS) could ultimately result in a progressive worsening of heart function, which may lead to signs of heart failure [74]. Finally, NPs affect normal cardiac development, as shown in studies on human pluripotent stem cells where exposure caused the formation of immature cardiomyocytes and organoids with decreased contractility, indicating potential risks to fetal and embryonic heart health [78].

Overall, evidence suggests that MNPs could be a new and significant environmental risk factor for myocardial dysfunction, cardiac fibrosis, arrhythmia, and heart failure through mechanisms such as oxidative stress, chronic inflammation, cellular aging, and ongoing mitochondrial damage.

## 4. Cardiovascular Toxicity Mechanisms

MNPs induce cardiotoxicity through a complex and highly interconnected network of molecular and cellular mechanisms that should be interpreted as a unified pathological cascade rather than isolated events (Table 1). Current evidence indicates that mitochondrial dysfunction and oxidative stress could represent primary upstream drivers, which initiate and propagate downstream inflammatory signaling, ion channel dysregulation, and multiple forms of regulated cell death, finally converging toward myocardial injury and cardiovascular dysfunction.

**Figure 3:**
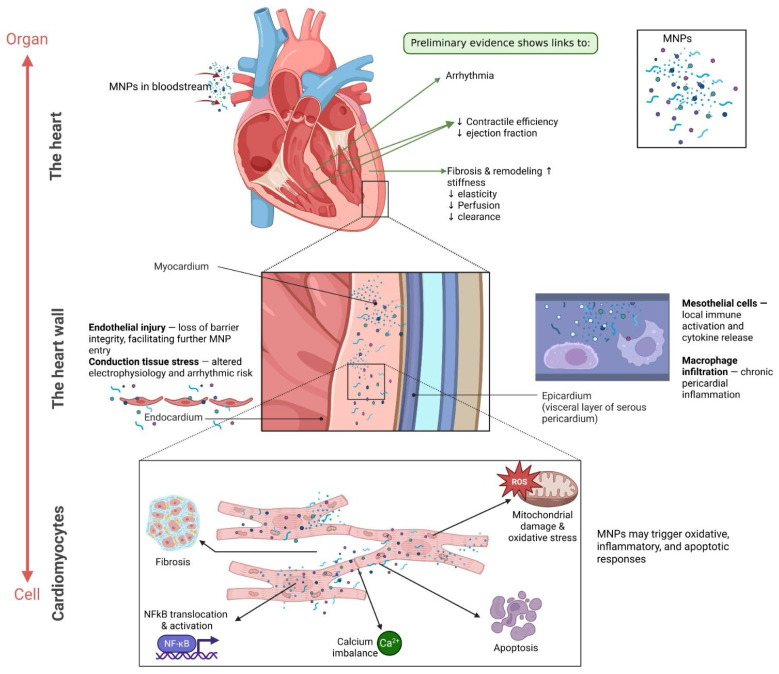
Multiscale effects of micro- and nanoplastics (MNPs) on cardiac health. At the organ level, circulating MNPs may accumulate in the myocardium and disrupt electrical conduction and excitation–contraction coupling, potentially contributing to arrhythmias and reduced contractile efficiency (e.g., decreased ejection fraction). In parallel, inflammation-driven remodeling may increase stiffness and reduce elasticity and perfusion, impairing overall cardiac function. At the tissue level, MNPs may compromise endothelial barrier integrity, facilitating further particle entry and vascular dysfunction. In the epicardial region, MNP exposure may activate mesothelial cells and promote cytokine release, supporting macrophage infiltration and chronic inflammation. These processes may enhance fibrosis and structural remodeling, disrupting myocardial architecture and conduction pathways. At the cellular level, internalized MNPs may induce mitochondrial dysfunction and reactive oxygen species (ROS) generation, impairing ATP production. Oxidative stress may activate inflammatory signaling (e.g., NF-κB) and disturb calcium handling, contributing to ion imbalance and defective contraction. These combined effects may promote apoptosis and fibrotic responses, ultimately leading to cardiomyocyte dysfunction.

In fact, current evidence indicates that MNPs exert different toxic effects at the cardiovascular level: nucleic acid alterations, cytotoxicity, immunotoxicity, and neurotoxicity. Additionally, they may disrupt cell metabolism and trigger inflammation. Together, these effects have varying severity across cardiovascular diseases, including atherosclerosis, cardiomyopathies, electrical alterations, and abnormalities related to development, congenital defects, and aging (Figure 3).

### 4.1. Integrated Oxidative, Inflammatory, and Cell Death Mechanisms Underlying MNP-Induced Cardiotoxicity

Accumulating evidence indicates that MNPs induce cardiotoxicity through a strongly interconnected network of molecular and cellular mechanisms centered on oxidative stress and mitochondrial dysfunction [12]. Following internalization into cardiomyocytes or endothelial cells or entry into the myocardial microenvironment, MNPs disrupt mitochondrial respiration and antioxidant defense systems, leading to the excessive production of reactive oxygen species (ROS) and loss of mitochondrial membrane potential [88].

Mitochondrial damage and sustained oxidative stress promote the release of damage-associated molecular patterns (DAMPs), which activate innate immune signaling pathways, particularly the NLRP3 inflammasome complex [89]. Inflammasome activation leads to caspase-1 cleavage and the secretion of pro-inflammatory cytokines, including interleukin-1β and interleukin-18, resulting in macrophage recruitment and the amplification of local inflammatory responses [90]. This persistent inflammatory microenvironment stimulates cardiac fibroblasts to overexpress pro-fibrotic genes, promoting excessive collagen synthesis and extracellular matrix deposition. Consequently, myocardial stiffening, impaired relaxation, and pathological ventricular remodeling develop, ultimately contributing to reduced contractile performance and increased susceptibility to heart failure [91].

In parallel, oxidative stress and mitochondrial dysfunction disrupt intracellular iron homeostasis and activate stress-responsive signaling pathways that promote regulated cell death. MNP-induced mitochondrial injury facilitates iron release and lipid peroxidation, creating conditions favorable for ferroptosis. This process is further amplified by the activation of the hypoxia-inducible factor-1α (HIF-1α)/heme oxygenase-1 (HO-1) pathway, which increases intracellular Fe^2+^ levels beyond ferritin storage capacity, thereby accelerating oxidative damage [92]. Pharmacological inhibition of iron overload or HIF-1α signaling partially restores cell viability, underscoring the central role of iron-dependent mechanisms in nanoplastic-induced cardiotoxicity [93].

Moreover, excessive ROS accumulation and mitochondrial impairment are associated with ER stress and the activation of the unfolded protein response. Prolonged ER stress promotes apoptotic signaling through the upregulation of p53 and the activation of caspase-3 while simultaneously enhancing ferroptotic susceptibility by suppressing anti-ferroptotic proteins. These ROS-dependent ER stress pathways facilitate the coordinated activation of apoptosis and ferroptosis, contributing to progressive cardiomyocyte loss. In addition, inflammasome activation may promote pyroptotic cell death, and emerging evidence suggests that the combined activation of apoptotic, ferroptotic, and pyroptotic pathways may culminate in panoptotic responses under sustained MNP exposure [93].

Collectively, these interconnected mechanisms converge to exacerbate endothelial dysfunction, vascular remodeling, and myocardial injury. In experimental models, MNP exposure is associated with an increased oxidative burden, impaired mitochondrial dynamics, enhanced inflammatory signaling, and the widespread activation of regulated cell death pathways [32]. These processes promote cardiomyocyte depletion, fibrotic remodeling, and the deterioration of myocardial compliance, leading to ventricular stiffening and impaired systolic and diastolic function.

Overall, MNP-induced cardiotoxicity arises from an integrated molecular cascade in which oxidative stress and mitochondrial dysfunction act as primary upstream drivers linking inflammasome-mediated inflammation, iron-dependent ferroptosis, ER stress, and apoptotic signaling. The convergence of these pathways sustains chronic inflammation, promotes myocardial fibrosis, and accelerates pathological ventricular remodeling, ultimately contributing to the progression of cardiovascular disease [92].

### 4.2. Oxidative and ER Stress-Mediated Ion Channel Dysregulation by MNPs Causing Cardiac Arrhythmias and Necrosis

Building on this mechanistic framework, mitochondrial dysfunction and ROS generation also play a central role in altering cardiac electrophysiology. Alterations in ion channels caused by MNPs represent a downstream consequence of oxidative and ER stress, ultimately leading to electrophysiological dysfunction and cardiac arrhythmias. MNP exposure results in intracellular buildup within human cardiomyocytes, where these particles interact with transmembrane calcium (Ca^2+^) and potassium (K^+^) ion channels. This buildup triggers oxidative stress and ER stress, resulting in protein folding issues. These conditions promote conformational changes and the phosphorylation of voltage-gated ion channels, which then cause variability in action potential duration and instability in cardiac rhythm [71]. Zhang and collaborators [87] demonstrated in advanced cardiac organoid-on-a-chip models that MNPs disrupt calcium homeostasis and mitochondrial function, thereby diminishing the cells’ capacity to regulate excitation–contraction coupling. The accumulation of ROS and decreased mitochondrial efficiency impair the function of Ca^2+^ pumps and ion channels, leading to spontaneous calcium oscillations and irregular contractions [94].

Accumulation of MNPs may also contribute to arrhythmogenesis through interactions with interorgan signaling pathways. Specifically, the excessive production of the cytokine FGF21 by the liver can modify dehydrogenase activity, thereby affecting ATP availability for ion pumps [95]. Overall, oxidative damage and ER stress disrupt the synthesis and structural integrity of ion channels, while mitochondrial dysfunction reduces cellular energy availability, collectively resulting in Ca^2+^ dysregulation, asynchronous contractions, and increased arrhythmic risk. Furthermore, mitochondrial and energy dysfunctions further diminish the efficiency of ion pumps. Additionally, altered interorgan signaling can modify the redox environment and cardiac function in regions where MNPs accumulate. These findings highlight that MNPs act not only as direct cellular toxicants but also as systemic modulators of cardiac electrophysiology within the broader mitochondrial–ROS-driven pathogenic cascade.

### 4.3. Immune and Inflammatory Mechanisms Triggered by MNPs in the Myocardium

In parallel with these processes, mitochondrial damage and ROS generation contribute to the activation of both innate and adaptive immune responses within the myocardium. The presence of MNPs in coronary blood and myocardium has been detected, indicating a local exposure capable of triggering immune responses in the cardiac microenvironment [89]. The first contact of the immune system with MNPs leads to the activation of innate immunity mechanisms: monocytes and dendritic cells internalize MNPs, releasing pro-inflammatory cytokines [96]. This may contribute to chronic mononuclear myocarditis, which can lead to dilated cardiomyopathy.

At the molecular level, MNP-induced signals converge on the activation of the NLRP3 (NLRP3-ASC-caspase-1) inflammasome, which promotes the maturation of IL-1β and IL-18, thereby maintaining a self-perpetuating local inflammatory circuit [89]. This innate response is followed by a type of adaptive immune response: studies demonstrate that following exposure to MNPs in human PBMCs, the levels of regulatory T cells and Th2 cells are reduced, favoring Th1 and Th17 cells and increasing cytokine release, leading to an inflammatory phenotype [89]. Furthermore, inflammatory signals induced by MNPs can be disseminated systemically via extracellular vesicles (EVs): these vesicles can transport MNPs or bioactive contents, such as pro-inflammatory miRNAs, capable of reshaping the gene expression profile in target cells. This process can lead to the reprogramming of immune-inflammatory pathways and the distant transfer of activating signals, affecting the cardiovascular system [97]. In parallel, MNPs induce oxidative stress and increased ROS, which amplify the immune response by activating the inflammasome, activating NF-κB, and contributing to the Th1/Th2 imbalances observed in vivo, thereby promoting the release of inflammatory cytokines and maintaining myocardial inflammation [98]. Together, these immune mechanisms integrate with mitochondrial dysfunction and oxidative stress, contributing to a self-perpetuating cycle of inflammation, cellular damage, and progressive cardiovascular pathology.

## 5. Clinical Implications

### 5.1. Endothelial Damage and Vascular Dysfunction

The endothelium, a monolayer of cells lining blood vessel walls and the endocardium, performs functions such as regulating vascular tone, promoting angiogenesis, managing hemostasis, and providing antioxidant, anti-inflammatory, and antithrombotic effects. Endothelial dysfunction is a primary indicator of cardiovascular disease. MNPs reach blood vessels and either adhere to or are taken up by ECs. Their accumulation causes elevated ROS levels and oxidative stress [15]. Excess ROS react with NO produced by ECs, impairing platelet aggregation and leukocyte adhesion, which disrupts vascular function. Additionally, ROS activate transcription factors that induce inflammatory pathways [12]. At the same time, MNPs increase the expression of leukocyte adhesion molecules, weaken junctions between endothelial cells, and raise endothelial barrier permeability [74]. These events lead to reduced endothelium-dependent vasodilation, promote vasoconstriction, and create a prothrombotic state marked by increased platelet aggregation [6]. As a result, this cascade contributes to pathological processes such as atherogenesis, thrombosis, and chronic inflammation within the vessel wall. These findings align with ex vivo human studies and clinical data, where MNPs have been detected in cardiovascular tissues such as carotid plaques, thrombi, and heart tissue. Furthermore, their presence within plaques correlates with a higher risk of heart attack, stroke, and increased mortality [74]. However, it is important to note that current clinical evidence remains largely observational, and the detection of MNPs in cardiovascular tissues does not establish a direct causal relationship. These associations may reflect co-exposure to other environmental pollutants or underlying disease processes rather than a primary etiological role of MNPs.

Moreover, MNPs promote endothelial damage through ROS and inflammation, weaken tight junctions, and alter vasomotor signaling and coagulation. This could lead to vascular dysfunction (reduced vasodilation, increased vasoconstriction, a procoagulant state, and increased permeability), which accelerates atherosclerosis and thrombosis [28]. However, environmental co-contaminants commonly associated with MNPs, such as heavy metals, persistent organic pollutants, and endocrine-disrupting chemicals, may act synergistically, further complicating the attribution of specific cardiovascular effects to MNP exposure alone [99,100].

### 5.2. Contribution to Atherosclerosis and Arterial Stiffness

When MNPs interact with the endothelium, they stimulate the release of ROS and cause mitochondrial dysfunction. These processes activate pro-inflammatory signaling pathways and increase vascular permeability, facilitating leukocyte entry and adhesion. Disruption of the endothelial barrier allows lipoproteins and low-density lipoproteins to pass through and deposit, promoting the formation of atherosclerotic plaques [12]. In macrophages, MNPs interfere with lipid metabolism by upregulating receptors that boost the uptake of oxidized low-density lipoprotein. This lipid buildup transforms macrophages into foam cells, which, upon cell death, contribute to the development of the necrotic core within the plaque [5]. Clinical studies show that the presence of MNPs in carotid plaques is linked to a fourfold increase in the risk of cardiovascular events [33]. This link is due to MNPs’ ability to sustain an inflammatory microenvironment and oxidative stress, promoting plaque instability and acute thrombotic events. These findings are supported by research indicating that MNP levels in coronary blood and thrombi from patients with myocardial infarction correlate with increased pro-inflammatory cytokine production and macrophage infiltration [13]. MNPs enhance macrophage infiltration, leading to the release of proteolytic enzymes and procoagulant factors that drive plaque growth, inflammation, instability, and recurrence [13]. Nevertheless, such correlations may also be influenced by underlying systemic inflammation, comorbidities, or concurrent exposure to other environmental and lifestyle-related risk factors. Further research has explored the active biological role of MNPs within the coronary blood and thrombi [33]. These findings suggest that MNPs are not just passively trapped within clots or in contact with inflammatory cells but actively participate in biological processes. MNPs promote macrophage infiltration, leading to the release of proteolytic enzymes and procoagulant factors that cause plaque growth, inflammation, instability, and recurrence. Experimental evidence shows that MNPs induce endothelial aging, increase collagen synthesis, and reduce elastin content. These changes cause vessel stiffness, fibrosis, and reduced elasticity. Stiffened vessels experience higher hemodynamic stress on plaques, which promotes microtears, inflammation, and thrombosis [5]. In summary, MNPs contribute to four main pathogenic mechanisms: endothelial dysfunction, inflammation with foam cell formation, thrombogenesis, fibrotic remodeling, and arteriosclerosis with reduced elasticity of the vessel wall, influencing blood pressure. This offers a biologically plausible explanation for the observed link between MNP presence, increased atherosclerosis, and arterial stiffness. While these mechanisms provide biological plausibility for a contributory role of MNPs, further longitudinal and controlled studies are required to establish a causal relationship.

### 5.3. Potential Role of MNPs in Cardiomyopathies

MNPs represent an emerging class of environmental cardiotoxins capable of perturbing myocardial structure and function (cardiomyopathies). It has been demonstrated that polyethylene and PVC particles are detectable in human myocardial tissues, including atrial and ventricular cardiomyocytes, vessels, left atrial appendages, the pericardium and pericardial adipose tissue. It has been shown to have a close correlation with increased incidence of major adverse cardiovascular events, including a severe decrease in the ejection fraction, myocardial infarction, stroke and impaired repair [6]. These findings, also supported by animal studies, suggest that the myocardium constitutes both a target and a reservoir for circulating MNPs [12]. MNPs have also been linked to cardiomyocyte dysfunction, suggesting possible contributions to cardiomyopathies and arrhythmias [11]. For instance, exposure of neonatal ventricular myocytes to NPs has been shown to markedly reduce intracellular Ca^2+^ levels, mitochondrial membrane potential, and cellular metabolism, ultimately leading to decreased contractile force in cardiomyocytes [79]. In rat models, MNP exposure has been associated with cardiac fibrosis driven by the activation of the Wnt/β-catenin pathway and increased cellular apoptosis. Similarly, in vivo exposure to PS-NPs has been shown to elevate cardiac injury markers such as troponin I and creatine kinase-MB (CK-MB), disrupt mitochondrial DNA integrity, and activate the cGAS-STING signaling pathway, leading to myofibrillolysis, cell swelling, and cardiomyocyte apoptosis [17]. Furthermore, rats exposed to environmentally relevant concentrations of MNPs exhibited significantly elevated cardiac biomarkers for hypertrophy with increased interventricular septal thickness [11]. Further crucial depth comes from Liu and collaborators [95], who studied the organelle-specific toxicity of PVC-NPs in mouse heart cells. PVC particles mainly gathered in lysosomes and mitochondria, disrupting autophagic flow and oxidative phosphorylation. This dual organelle malfunction led to a significant drop in mitochondrial ATP production, a collapse in membrane potential, and severe cristae disorganization, ultimately resulting in heart cell death. In live subjects, long-term exposure to PVC-NPs caused bradycardia, sarcomere disarray, mitochondrial swelling, and interstitial fibrosis, reproducing key structural and functional features of human heart disease [83]. At the systemic level, MNP accumulation provokes chronic low-grade inflammation, oxidative stress, and cellular senescence in endothelial and immune cell pathways intimately linked to myocardial remodeling and heart failure [6]. The activation of NADPH-oxidase and NF-κB signaling, coupled with increased interleukin-1β and tumor necrosis factor-α expression, creates a pro-oxidant milieu that exacerbates mitochondrial injury and calcium mishandling within cardiomyocytes [101]. Collectively, these studies describe a convergent pathophysiological cascade, where MNPs penetrate cardiac tissues, accumulate within mitochondria and lysosomes, trigger oxidative and inflammatory signaling, and ultimately drive apoptosis, fibrosis, and contractile impairment. The current evidence defines a mechanistic continuum linking environmental MNP exposure to cardiomyocyte dysfunction and death, as well as fibrotic repair and progressive myocardial remodeling. These findings raise serious concerns and emphasize the urgent need for further investigation into MNP-induced cardiomyopathies [102,103]. Importantly, many of these findings derive from experimental models employing exposure levels that may exceed those currently detected in human tissues, highlighting a key limitation in translating these results to clinical risk. Additionally, co-exposure to other environmental stressors and individual susceptibility factors may significantly influence cardiovascular outcomes. Robust clinical evidence demonstrating causality is still lacking, underscoring the need for well-designed epidemiological and longitudinal studies.

## 6. Therapeutic Perspectives and Prevention Strategies

The toxic effects induced by MNPs highlight the urgent need for both therapeutic and preventive strategies. Future research should focus on clearly defined and testable priorities aimed at advancing mechanistic understanding and clinical translation. The combined human, animal, and cellular data indicate that MNPs may serve as novel environmental risk factors for cardiomyopathy, contributing to both the initiation and exacerbation of cardiac disease. This concept requires targeted translational studies integrating epidemiological cohorts with human cardiac organoids to quantify exposure thresholds, establish dose–response relationships, and identify causal pathways. In particular, a major research opportunity lies in defining the long-term effects of chronic low-dose exposure at physiologically relevant concentrations and identifying susceptible populations. Future research should prioritize the validation of antioxidant and mitochondrial-protective strategies (e.g., SOD mimetics and polyphenols) in human-relevant models, with particular attention to efficacy, dosing, and long-term safety [104,105].

Furthermore, the modulation of the NLRP3/caspase-1 inflammatory axis, activated upon MNP exposure, could represent another therapeutic avenue to limit cardiac fibrosis and structural remodeling [106]. A key opportunity is to determine the relative contribution of this pathway to disease progression and its suitability as a therapeutic target. At the cellular level, regulating autophagic flux and maintaining lysosomal-mitochondrial communication may hold therapeutic potential. Pharmacological activation of mitophagy or AMPK-mTOR signaling could therefore help restore cardiomyocyte homeostasis and preserve cardiac performance under MNP stress [83]. Future studies should systematically investigate these pathways across different MNP physicochemical properties (size, shape, and polymer composition) to establish structure–toxicity relationships.

Another major research priority is the development of reliable biomarkers of exposure and early cardiac injury. The presence of plastic polymers in human atherosclerotic plaques and cardiac tissues suggests that chronic, low-level exposure may already be contributing to cardiovascular morbidity at a population level. This supports the need for standardized and sensitive detection techniques applicable in both clinical and environmental settings. The identification of circulating or imaging-based biomarkers represents a critical step toward integrating MNP exposure into cardiovascular risk assessment. Finally, interdisciplinary research integrating cardiovascular medicine, toxicology, and materials science should aim to define safe exposure thresholds and develop technologies to reduce MNP bioavailability. Overall, future research should adopt a multiscale and translational framework to define risk, identify therapeutic targets, and support the development of effective mitigation strategies.

## 7. Conclusions

The toxic effects induced by MNPs highlight the urgent need for both preventive strategies and therapeutics. When these particles enter the body or reach the heart through the bloodstream, they trigger various molecular and cellular responses that damage the heart’s structure and function. These effects range from the activation of the immune system, evidenced by inflammasome activation and cytokine release, to mitochondrial dysfunction, increased oxidative stress, and cell death through apoptosis and ferroptosis. Their effect on ion channels and contraction–relaxation processes leads to electrical disturbances and arrhythmias. Moreover, their buildup in endothelial cells results in oxidative and inflammatory damage, vascular issues, and atherothrombotic events. Together, these findings show that MNPs are not only local toxins but also systemic stressors that can disrupt the delicate balance of heart and vascular health. Despite these insights, many important questions remain unanswered. Several limitations should also be acknowledged. Much of the current evidence is derived from in vitro and animal models, which may not fully recapitulate human cardiovascular physiology, and the heterogeneity in particle types, exposure conditions, and experimental designs complicates direct comparisons across studies. In addition, discrepancies between the concentrations used experimentally and those detected in human tissues may limit immediate clinical translation, while the lack of standardized detection and quantification methods remains a significant challenge in the field. Nevertheless, these limitations also reflect the emerging and rapidly evolving nature of MNP research, underscoring the importance of integrative frameworks such as the one proposed here to consolidate existing knowledge, identify critical gaps, and guide future mechanistic and translational investigations.

Furthermore, we still do not fully understand the dose–response relationship, the effects of long-term buildup, or how MNPs interact with other environmental or metabolic stressors. Additionally, translating experimental results to human health requires thorough investigations in living organisms and clinical settings. Innovative in vitro models, such as human iPSC-derived cardiac organoids and organ-on-a-chip systems, could provide promising ways to explore how MNPs cause heart damage under more realistic conditions. In summary, MNPs represent a new group of environmental pollutants that harm cardiovascular health, leading to an increased risk of cardiomyopathy. Future research that combines toxicology, epidemiology, cardiovascular science, and materials science will be crucial for fully understanding their effects and developing strategies to reduce the cardiovascular risks linked to micro- and nanoplastic exposure.

## Figures and Tables

**Table 1:** Experimental and clinical evidence linking micro- and nanoplastics (MNPs) to cardiomyopathy and cardiac dysfunction.

Model/Study Type	MNPs Type and Exposure	Possible Cardiac Effects	Mechanisms Involved	Reference
**In vitro (neonatal ventricular myocytes)**	Polystyrene nanoparticles (1–100 nm)	Decreased intracellular Ca^2+^, mitochondrial membrane potential, and contractile force	Mitochondrial dysfunction and metabolic impairment	[79]
**In vivo (rat model)**	Polystyrene microplastics (oral exposure)	Cardiac fibrosis, apoptosis, and electrical abnormalities	Activation of Wnt/β-catenin and NLRP3/caspase-1 pathways	[80]
**In vivo (rat, human-equivalent dose)**	Mixed MNPs (polystyrene, polyethylene)	Elevated troponin I and CK-MB; increased septal thickness	Mitochondrial DNA damage and activation of the cGAS–STING pathway	[81,82]
**In vitro (HL-1 cardiomyocytes)**	PVC nanoplastics (1–100 nm; 1–5 µg/mL)	Decreased ATP levels and mitochondrial potential; vacuolization	Lysosomal and mitochondrial dysfunction; impaired autophagic flux	[83]
**In vivo (mouse, 4-month exposure)**	PVC nanoplastics (oral gavage)	Bradycardia, sarcomeric disarray, interstitial fibrosis	Mitochondrial cristae loss and oxidative stress	[83,84]
**In vitro (endothelial cells)**	Polystyrene nanoplastics	Increased ROS generation, apoptosis, and endothelial dysfunction	NADPH oxidase activation and p53 upregulation	[85]
**Human studies**	Polyethylene and PVC identified in carotid plaques, myocardium, and thrombi	Increased risk of myocardial infarction, stroke, and all-cause mortality	Chronic inflammation, oxidative stress, and immune activation	[86,87]

## Data Availability

No new data were created or analyzed in this study. Data sharing is not applicable to this article.
